# Frobenius-norm-based measures of quantum coherence and asymmetry

**DOI:** 10.1038/srep32010

**Published:** 2016-08-25

**Authors:** Yao Yao, G. H. Dong, Xing Xiao, C. P. Sun

**Affiliations:** 1 Microsystems and Terahertz Research Center, China Academy of Engineering Physics, Chengdu, Sichuan 610200, China; 2 Institute of Electronic Engineering, China Academy of Engineering Physics, Mianyang, Sichuan 621999, China; 3 Beijing Computational Science Research Center, Beijing, 100193, China; 4 College of Physics and Electronic Information, Gannan Normal University, Ganzhou, Jiangxi 341000, China

## Abstract

We formulate the Frobenius-norm-based measures for quantum coherence and asymmetry respectively. In contrast to the resource theory of coherence and asymmetry, we construct a natural measure of quantum coherence inspired from optical coherence theory while the group theoretical approach is employed to quantify the asymmetry of quantum states. Besides their simple structures and explicit physical meanings, we observe that these quantities are intimately related to the purity (or linear entropy) of the corresponding quantum states. Remarkably, we demonstrate that the proposed coherence quantifier is not only a measure of mixedness, but also an intrinsic (*basis-independent*) quantification of quantum coherence contained in quantum states, which can also be viewed as a normalized version of Brukner-Zeilinger invariant information. In our context, the asymmetry of *N*-qubit quantum systems is considered under local *independent* and *collective* 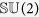 transformations. In- triguingly, it is illustrated that the collective effect has a significant impact on the asymmetry measure, and quantum correlation between subsystems plays a non-negligible role in this circumstance.

In the 20^th^ century, quantum mechanics, as the core of quantum physics, is undoubtedly one of the most profound scientific theories during the development process of modern science and philosophy. Notably, quantum coherence is one of the most remarkable and characteristic traits of quantum mechanics and also viewed as the critical resource for the emerging field of quantum technologies, such as quantum cryptography^1^, quantum metrology^2^ and quantum computation^3^. This intrinsic principle of quantum mechanics enforces its entire departure from classical lines of thought, similar to the notion of quantum entanglement^4^. In different occasions, the generalized concept of quantum coherence manifests itself as quantum superposition, quantum asymmetry or non-commutability. These specific notions have gotten extensive applications in quantum physics both theoretically and experimentally. Therefore, the characterization, quantification and application of quantum coherence are one of the central topics in quantum information science.

Although the investigation of coherence theory has a long history in classical and quantum optics^5^,^6^,^7^,^8^,^9^, it does not provide an integrated and unified framework for quantitative study. In some contexts, even conflicting definitions have appeared with respect to the same concept^10^,^11^,^12^,^13^,^14^,^15^, especially when the vectorial character of electromagnetic waves is involved. From the perspective of quantum information theory, a rigorous framework for the characterization and quantification of coherence has been formulated very recently, based on the quantum resource theory^16^,^17^. On one hand, Baumgratz *et al*. defined the incoherent states and incoherent operations and further discussed the quantum coherence monotones in the constraint of a series of axiomatic-like prerequisites^18^, which is extremely similar to the approach adopted in quantum entanglement theory^19^. Within this framework, novel measures of quantum coherence have been proposed and the connections between coherence and other manifestations of quantum correlations have been carefully scrutinized^20^,^21^,^22^,^23^,^24^,^25^,^26^,^27^,^28^,^29^,^30^,^31^. On the other hand, Marvian and his collaborators proposed the resource theory of asymmetry which included the former notion of quantum coherence as a special case^32^,^33^,^34^,^35^,^36^,^37^. Moreover, the internal relations between these two approaches and their physical justifications have been further clarified lately^38^,^39^.

However, the story has never come to the end. The investigation of quantum coherence is now facing several crucial problems that need to be solved urgently. First of all, we should further identify the application scopes and physical meanings of existing measures of quantum coherence. In particular, the quantifiers presented in ref. 18 are established in the finite-dimensional setting and thus can not be directly applied to the infinite-dimensional systems. Moreover, explicit physical (or operational) meanings of existing measures of quantum coherence are to be pursued by the community, though partial results have been obtained^31^,^40^,^41^. Secondly, as indicated by Marvian *et al*., distinct approaches of quantifying quantum coherence will lead to different conclusions in a diverse set of tasks. For instance, the *speakable* notions of coherence may not be suitable to characterize the consumed resource in the task of phase estimation^37^,^38^. Moreover, Chitambar *et al*. also pointed out that all currently proposed *basis-dependent* theories fail to satisfy a criterion of physical consistency^39^. In addition, the interactions between quantum coherence and other manifestations of quantum correlations still need to be uncovered^22^,^23^. Finally, the quantitative relations between quantum coherence and other important quantities in quantum information are yet to be established^42^,^43^.

In contrast to the resource-theory framework, we notice that other types of coherence or asymmetry measures are also of great significance in optical coherence theory and condensed matter physics. For example, a measure of *first-order coherence* 
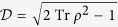
 (for qubit subsystem) was exploited to introduce the concept of accessible coherence^44^ and it was recently demonstrated that 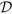 can be unveiled from hidden coherence in partially coherent light^45^. Moreover, Fang *et al*. proposed a novel measure of the degree of symmetry by virtue of group theoretical approach and showed that this quantity can effectively detect the phenomena of accidental degeneracy and spontaneous symmetry breaking^46^. In this work, we formulate two Frobenius-norm-based measures by further extending their previous work and more importantly, illustrate the clear physical meanings of these quantifiers. Through the establishment of relationship with other significant physical quantities (e.g., Brukner-Zeilinger information), we emphasize that the purity of quantum states is not only a measure of mixedness, but also a *basis-independent* quantification of quantum coherence contained in quantum states.

## Results

### Coherence measure based on Frobenius norm

In the famous work of Mandel and Wolf, they introduced an important measure depicting the *degree of polarization* for planar electromagnetic fields^7^


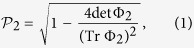


where Φ_2_ represents 2 × 2 equal-time coherence matrix at a given point 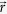 and 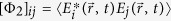, *i*, *j* = *x*, *y*. Note that this quantity characterizes the coherence between two mutually orthogonal components of the complex electric vector ***E***(*t*) = [*E*_*x*_(*t*), *E*_*y*_(*t*)]*T*. Be aware of the Hermiticity and non-negativity of the matrix Φ_2_, we can define a physically valid quantum state *ρ* = Φ_2_/TrΦ_2_. Since the Bloch sphere is analogous to the Poincaré sphere, 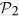 can be rewritten as


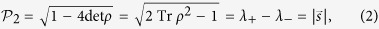


where we have used the Bloch representation 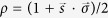 and 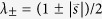 are eigenvalues of *ρ*. It is apparent that 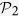 is equivalent to the degree of first-order coherence 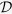 adopted in refs 44,45 and exactly equal to the length of the Bloch vector 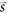. In fact, in this case (e.g., for qubit system) *ρ* can also be recast as


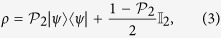


with 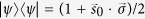 and 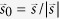.

Furthermore, Setälä *et al*. moved a step forward and extended the above 2D formalism into the formulation of the 3D degree of polarization coherence for arbitrary electromagnetic fields^47^,^48^, that is


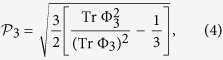


where Φ_3_ denotes the 3 × 3 coherence matrix at a given point 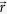 and 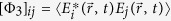, *i*, *j* = *x*, *y*, *z*. Similarly, by redefining *ρ* = Φ_3_/Tr Φ_3_, we obtain


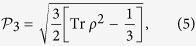


Actually, we observe that this formalism can be unified and generalized to arbitrary dimensional systems, motivated by the work of Luis^49^, where a 4 × 4 density matrix was considered. The key idea is to quantify quantum coherence from the geometrical point of view, that is, the degree of coherence between distinct variables of optical fields is assessed as the distance between the coherence matrix and the identity matrix. Note that this geometrical approach is also employed in quantum resource theories^16^,^17^,^18^,^19^. Here the identity matrix, or maximally mixed state in the sense of quantum information, is identified as the fully incoherent and completely unpolarized light^7^,^49^. Therefore, we can construct the following general measure


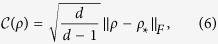


where *d* is the dimension of the Hilbert space, 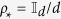 denotes the maximally mixed state. and the Frobenius norm is given by 
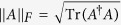
. Note that here the Frobenius norm is normalized to guarantee 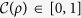 (see Methods section for more details). It is worth pointed out that this measure inherently possesses the following desirable properties:

- When *d* = 2, 3, 4, the coherence measure^6^ reduces to the existing quantifiers for 2D, 3D and 4D cases, where for 4D formalism, this measure was also termed as the *depolarization index*^50^,^51^.
- No optimization is involved in 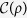, which is in sharp contrast to the coherence measures proposed in ref. 18, though the optimization is rather straightforward there.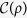 is independent of the specific representation of *ρ*, that is, 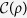 is *basis-independent*.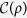 is unitarily invariant, since 
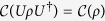
 owing to the fact that the maximally mixed state 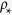 is *the only* state that remains invariant under arbitrary unitary transformation. In fact, the property (iv) is equivalent to (iii).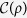 has an analytical expression and explicit geometrical interpretation. As we shall see below, 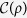 also has a clear operational meaning.

To further elaborate on the characteristics of 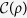, we first present several observations where this coherence measure is involved.

*Observation I*: Straightforward calculation shows that the square of 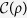 is directly proportional to the celebrated *Brukner-Zeilinger invariant information*^52^


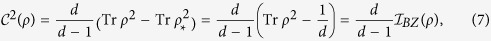


where the Brukner-Zeilinger information 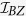 is an operational notion defined as the sum of individual measures of information over a complete set of mutually complementary observables (MCO)^53^


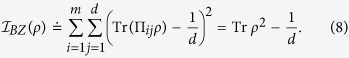


Here *i* = 1, …, *m* and *j* = 1, …, *d* label complementary observables and their eigenvectors, respectively. A significant feature of 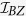 is its independence of the specific choice of the measured set of MCO and this independence is also equivalent to the unitary invariance of 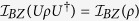, since the unitary transformations do not alter the eigenvalues of *ρ* and Tr*ρ*^2^ only relies on them. Therefore, it is evident that 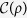 (and 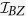) is a measure of *basis-independent* coherence contained in quantum states and quantifies the intrinsic randomness irrespective of the amount of entanglement between subsystems (if there are any). It is worth noting that the (global) purity does not solely determine the entanglement of a multi-partite state and this fact led to the investigation of the so-called *maximally entangled mixed states* for a given degree of purity^54^,^55^. Moreover, all pure states should always be represented as a coherent superposition of a certain set of basis states rather than a classical mixing, and by our definition 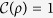 for any pure state. This is reminiscent of the argument by von Neumann that the entropy of all pure states is defined to be 0 as a kind of normalization^56^. Finally, since the Brukner-Zeilinger invariant information was successfully utilized in quantum teleportation^57^, state estimation^58^ and the violation of Bell’s inequalities^59^, 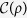, as a renormalized version of 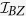, also plays an important role in all these quantum information tasks.

*Observation II:* The coherence measure 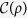 is associated with entropy production problem of doubly-stochastic (bistochastic) quantum channels. By use of the quantum version of Kullback inequality (or a stronger Pinsker inequality^60^)


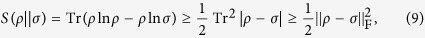


Streater proved the following theorem^61^:

**Theorem 1.** Let 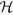 be a Hilbert space with dim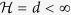 and denote by 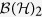 the Hilbert space of operator on 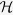 with scalar product 〈A, B〉 = Tr (A^†^B). If 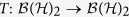 is a bistochastic channel which is ergodic and has spectral gap γ ∈ [0, 1) (e.g., up to a scalar multiple, the identity matrix 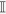 is the only fixed point of T in 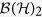, and the spectrum of the channel T^†^ ◦T is contained in the set [0, 1 − γ]∪{1}), then for any density matrix ρ


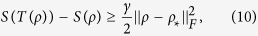


where *S*(*ρ*) = −Tr(*ρ* ln *ρ*) is the von Neumann entropy of *ρ*.

From Theorem 1, we find that two separated terms contribute to the lower bound of the entropy production. The first term 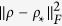 is just the unnormalized version of 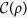, which characterizes the intrinsic coherence independent of the choice of a specific basis, while the spectral gap *γ* relies on representations of both *T* and *ρ*. To further elucidate the role of the bistochastic channel *T*, we consider the thermodynamics problem raised in ref. 62, where the the bistochastic channel was taken to be a projection measurement *ρ* → Δ(*ρ*) : = ∑_*k*_Π_*k*_*ρ*Π_*k*_. If {|*l*〉}_*l*_ is the eigenbasis of *ρ* and {Π_*k*_ = |*ϕ*_*k*_〉〈*ϕ*_*k*_|}_*k*_ constitute another orthogonal basis, then *γ* is the second smallest eigenvalue of the matrix 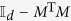, where *M* is a bistochastic matrix with the entries *M*_*kl*_ = 〈*l*|Π_*k*_|*l*〉 = |〈*ϕ*_*k*_|*l*〉|^2^. It is easy to show that *γ* is zero if {|*l*〉}_*l*_ and {|*ϕ*_*k*_〉}_*k*_ are the same, while *γ* = 1 if they are mutually unbiased. A deeper insight can be gained by noticing that once quantum thermodynamics is viewed as a resource theory, it can be cast as a *hybrid* of the resource theory of purity and the resource theory of asymmetry^16^. In particular, if {Π_*k*_}_*k*_ are the eigenvectors of an observable *L*, *γ* (e.g., second smallest eigenvalue of 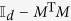) is a quantifier to characterize to what extent *ρ* fails to commute with *L* and *γ* ≠ 0 if and only if *ρ* has some coherence over the eigenspaces of *L*. This line of thought coincides with that of Marvian *et al*.^36^.

*Observation III:* For a square *d*-dimensional matrix *A* and *p* ∈ [1, +∞), the *l*_*p*_ norms (or vector norms) and Schatten-*p* norms are defined as^63^


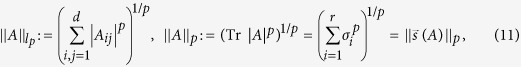


where 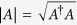, *r* = *rank*(*A*) and 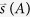 is used to refer to the vector of nonzero singular values 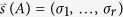. In addition to the monotonicity 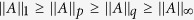 for 1 ≤ *p* ≤ *q* ≤ ∞, we can prove the following inequality by using Hölder’s inequality^64^


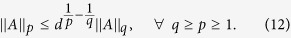


Note that the monotonicity and the above inequality^12^ is also satisfied by *l*_*p*_ norms, and the Schatten-*p* norms of *A* coincides with the ordinary (vector) *l*_*p*_ norms of 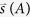. In particular, the Frobenius norm of *A* coincides with the corresponding *l*_2_ norm. Therefore, 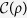 gives a upper bound of *l*_1_ norm coherence and trace-norm coherence discussed in refs 18 and 27 respectively


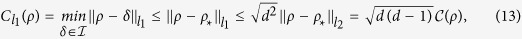



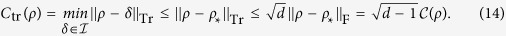


where 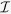 denotes the set of diagonal states defined in a pre-fixed basis. It is not surprising that the upper bounds 13^,^^14^ are not tight in general though 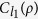 and *C*_tr_(*ρ*) indeed achieve their maximum values with the closest state 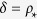. In fact, 
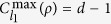
 and 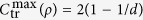, which is satisfied by the maximally coherent state^18^.

On the other hand, it is intuitive to assume that the intrinsic coherence measure 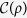 may have a natural relationship with the resource theory of purity^65^, due to the fact that 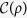 is proportional to 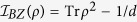. Actually, 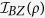 provide a lower bound for the so-called *unique measure of information* introduced in ref. 66. By virtue of the quantum Kullback inequality^9^, we have


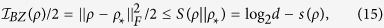


where 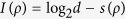 is a unique measure for information, in the sense that 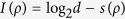 is equal to the optimal transition rate for mixed states to one qubit pure state *π* (i.e., *π* = |0〉〈0|)^16^,^65^. However, since the Kullback inequality is not very sharp, this lower bound is rather loose, especially for the states with high purity.

### Cohering (Purifying) power of quantum channels

With the framework of Baumgratz *et al*.^18^, we proposed the notion of cohering power of quantum channels (i.e., completely positive and trace preserving maps)^23^, which has been further explored in ref. 67. In the context of this work, we can introduce a similar quantity to characterize the cohering (purifying) capacity of quantum channels


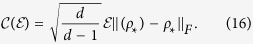


Complete positivity of 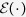 enables a Kraus decomposition


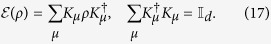


where the second relation stems from the trace preserving property. Thus, the cohering power^16^ can be rewritten as


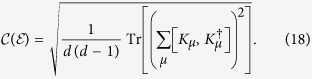


It is observed that the operator 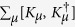 is Hermitian and traceless. Moreover, 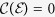 if and only if 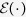 is a unital quantum channel, i.e., doubly-stochastic (bistochastic) completely positive map or 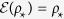
68. Therefore, the cohering capacity 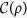 can be recognized as a measure of *nonunitality* of quantum channels. Note that the nonunital channels also play a crucial role in the local creation of quantum correlations^69^. Within the scope of resource theories^16^,^17^, we *may* make a correspondence in our context: the only free state is the maximally mixed state 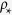, the free operations are unital channels and the resources are states with 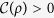. The justification of such a correspondence relies on the monotonicity of the function 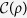 under unital channels. The following theorem proves that it is the case.

**Theorem 2** For any unital (i.e, bistochastic) quantum channel, it holds that 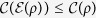.

*Proof*. Since 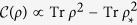, it is sufficient to prove 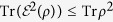. Consider the Kraus decomposition of 
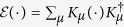
 and we have


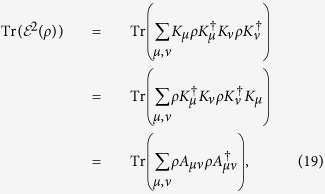


where we have defined 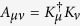. It is interesting to see that {*A*_*μν*_} also constitute a unital quantum channel 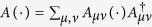 due to the identities


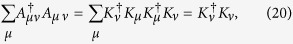



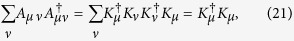


where the unitality of 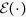 is used. With the aid of Chauchy-Schwarz inequality for operators 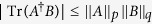 for *p*^−1^ + *q*^−1^ = 1 (e.g., *p* = *q* = 2 in our case), we finally obtain


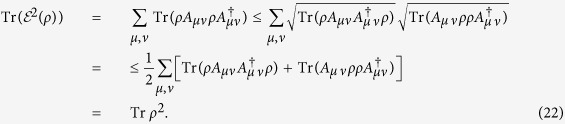


where the unitality property of *A*(⋅) is applied.

We have attempted to check whether the unitality of quantum channels is also a necessary condition for the monotonicity of 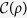. Note that for finite-dimensional Hilbert spaces, a dynamical semigroup is strictly purity-decreasing if and only if the Lindblad generator is unital^70^. However, actually the Kraus or operator-sum representation is more general than the master equation approach^3^. Therefore, the necessity of unitality of operator-sum representation for strictly-decreasing purity is still an important unsolved problem. See Method section for more information.

### Asymmetry measure based on Frobenius norm

In realistic physical scenarios, there are some states which are invariant under a set of symmetric operations or a given symmetric group. This fact tells us that a specific group of symmetric transformations assigns a specific type of symmetry to the underlying systems. A state *ρ* is called symmetric (or *G*-invariant) with respect to a finite or compact Lie group *G* if it satisfies^34^,^36^,^71^


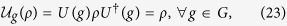


where *U*(*g*) denotes a unitary representation corresponding to the group element *g* ∈ *G*. We notice that this definition of symmetry is equivalent to other two criterions


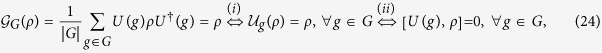


where the summation in 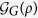 will be replaced by integral over *dg* when a compact Lie group is considered. The criterion (i) induced a nature entropic measure of the asymmetry of *ρ* with respect to *G*^36^,^71^, (i.e., 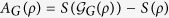), which was also proved to be a measure of the quality of a quantum reference frame^33^. Based on the criterion (ii), it is realized that the commutator [*U*(*g*), *ρ*] characterizes to what extent *ρ* is asymmetric with respect to *G* and some matrix norm of [*U*(*g*), *ρ*] should be a reasonable measure of *G*-asymmetry^36^.

Along this line of thought, a new quantifier of asymmetry has recently been proposed by Sun’s group, which is defined as an average of the *fidelity deviations* of Hamiltonian *H* (or quantum state *ρ*) over a specific symmetric group *g* ∈ *G*^46^


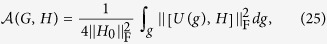


where *H*_0_ = *H* − Tr*H*/*d* is a re-biased version of *H*. 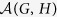 is mathematically tractable and also has desirable properties (e.g., basis-independence), mainly owing to the geometric feature of the Frobenius norm^64^. More importantly, it is has been demonstrated that 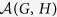 can effectively detect some significant phenomena in condensed matter physics, such as accidental degeneracy and spontaneous symmetry breaking^46^. Meanwhile, an analogous measure can be defined since we are more concerned with how to quantify the asymmetry (symmetry) of quantum states


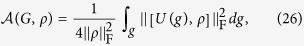



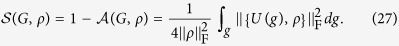


With this formulation, we can investigate the asymmetry of any quantum state with respect to a specific symmetric group. Remarkably, while seemingly unrelated, there actually exists a close connection between 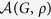 and 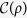. We first focus on the single qubit system 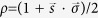, where 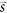 is the Bloch vector and can be parameterized as 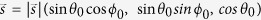 with polar and azimuthal angles (*θ*_0_, *ϕ*_0_). Since the group of all linear unitary operations over a single qubit is equivalent to a 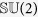 algebra, here what interests us most is to investigate the performance of 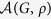 under the 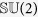 group. The group element of 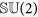 can be expressed as


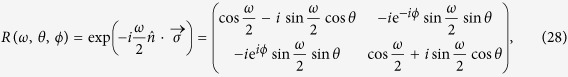


where 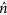 is a real unit vector and for simplicity we denote the triple as a vector 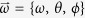. With the identity 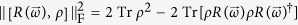 and 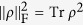, the quantity 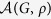 for 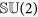 group can be explicitly calculated


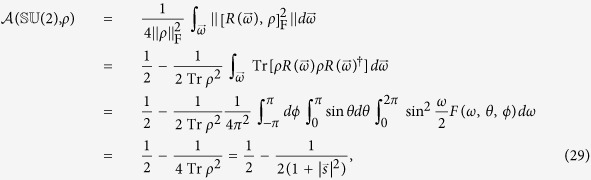


where we have used the integral formula for a functional 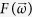 over the 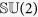 group^72^


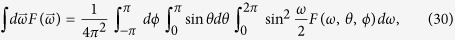


and in our case the functional 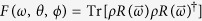 can be represented as


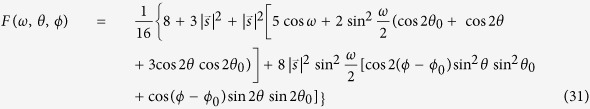


As expected, the result^29^ indicates that 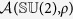 is a basis-independent quantity, that is, it does not rely on the initial orientation of 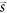, although *F*(*ω*, *θ*, *ϕ*) explicitly depend on *θ*_0_ and *ϕ*_0_. Moreover, when 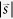 = 0, the initial state *ρ* is the maximally mixed state 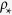 and meanwhile 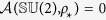. This observation coincides with the fact that for any symmetry and for any representation of the symmetry, the completely mixed state 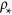 is invariant under all symmetry transformations^34^. Intriguingly, we build up a relationship between 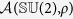 and 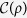 for arbitrary single-qubit system


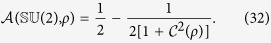


Apparently, 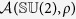 is a monotonic increasing function of 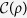, which implies that more intrinsic coherence signifies more asymmetry under the 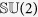 group. Such a simple but novel relation motivates us to inspect 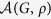 for *N*-qubit states by invoking the tensor product structure of Hilbert space.

An arbitrary *N*-qubit (pure or mixed) states can be written as


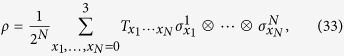


where 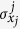 (*x*_*j*_ = 1, 2, 3) are the standard Pauli matrices in the Hilbert space of qubit *j* and 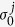 is the corresponding identity operator. The set of real coefficients 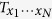 with *x*_*j*_ = 1, 2, 3 constitutes the so-called correlation matrix *T*. To gain further insight into the property of 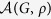, we consider the symmetry group 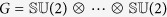, where the group element is 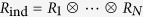 and 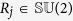 are *independent* local unitary operations acting on qubit *j*. Similar to the single-qubit case 29, the key procedure is to calculate the average of 
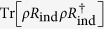
 over 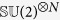. Due to the tensor product structure of N-qubit states and the trace formula Tr(*A*⊗*B*) = Tr(*A*)Tr(*B*), we have the relation


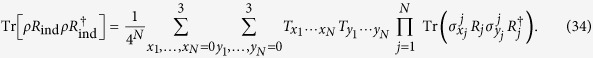


Therefore, the problem reduces to the evaluation of the integral 
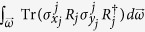
. In fact, we can prove that only when *x*_*j*_ = *y*_*j*_ = 0, this integral is nonzero, namely (see Methods section)


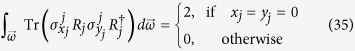


Finally, we obtain the asymmetry of *N*-qubit states under 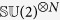 in a general form


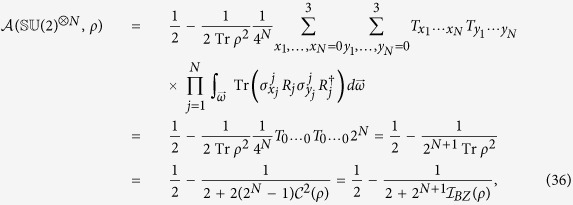


where the coefficient 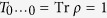. Obviously, this result reduces to the single-qubit case for *N* = 1 and the maximally mixed state 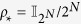 still corresponds to the minimum value of 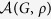 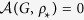. In addition to the unambiguous and monotonous relation between 
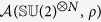
 and 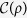 (hence 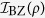), it is worthwhile to note that 
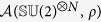
 is independent of the correlation matrix *T*, more precisely, independent of the internal quantum correlations (e.g., entanglement) between subsystems.

Intuitively, one might be tempted to conjecture that *any* local symmetric transformation group *G* will lead to a *correlation-independent* measure of asymmetry. However, this is not the case. To illustrate this, let us take a closer look at local unitary transformations. Other than the independent local unitary transformations considered above, their *collective* counterparts also plays a crucial role in quantum information and computation^73^. In this circumstance, the element of symmetry group is the tensor product of *N identical* unitary transformations 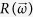, that is, 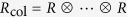. Take two-qubit states for example and we obtain (see Methods section for more details)


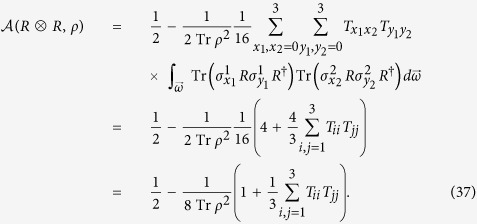


It is evident that the collective effect exhibits a significant influence on the asymmetry measure and 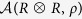 indeed relies on the diagonal entries of the correlation matrix *T*, which are basis-dependent quantities.

## Discussion

In this work, we first formulate a coherence measure 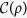 based on the Frobenius norm, which is defined from a geometric perspective. Remarkably, we have demonstrated that this quantity is not only a measure of mixedness, but also an intrinsic (*basis-independent*) quantification of quantum coherence contained in quantum states, which can also be viewed as a normalized version of Brukner-Zeilinger information^52^. To further illustrate this point, a comparison can be made between 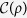 and the coherence measures proposed by Baumgratz *et al*.^18^. For example, for a single-qubit state 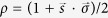, those quantifiers are listed as follows


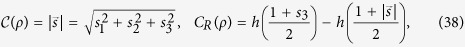



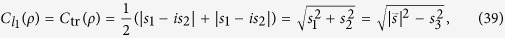


where *h*(*x*) = −*x*log *x* − (1 − *x*)log (1 − *x*) is the binary entropy function and *C*_*R*_(*ρ*) is the relative entropy of coherence. From the above expressions, it is evident that *C*_*R*_(*ρ*), 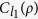 and *C*_tr_(*ρ*) are all basis-dependent measures, since they all depend on *s*_3_, which is not a unitarily-invariant quantity. Moreover, the participation of *s*_3_ precisely reflects the choice of the pre-fixed basis. In fact, we can present an alternative formulation of 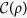 based on the eigenvalues *λ*_*j*_ of *ρ*, by noticing that *λ*_*j*_ − 1/*d* is the corresponding eigenvalues of 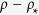, which is a Hermitian and traceless operator


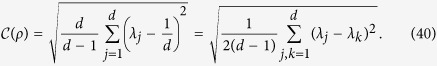


Note that 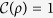 for any pure state. This result is just a consequence of the basis-dependent property of 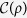: pure states can only be represented as a coherent superposition in *any* pre-fixed basis (without including itself as a base vector), and more precisely, any pure state can always be connected by a unitary transformation within a given dimension. In contrast to quantum correlations, 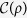 is a *global* property of quantum states, which quantifies the intrinsic randomness irrespective of the amount of entanglement between subsystems. This is reminiscent of the argument by von Neumann that the entropy of all pure states is defined to be zero as a kind of normalization, but the entropy itself does *not* give a clue on the quantum correlations between subsystems of the (pure) states. Moreover, the current construction of coherence measure is not valid for infinite dimensional systems and a possible solution is to invoke the so-called extended (or unitized) Hilbert-Schmidt algebra instead^74^.

Analogous to previous works^23^,^67^, the cohering power 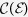 of quantum channels is introduced in our context. It is demonstrated that 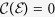 if and only if 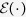 is a unital quantum channel, i.e., doubly-stochastic (bistochastic) completely positive map. Therefore, the cohering capacity 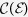 can be recognized as a measure of *nonunitality* of quantum channels. For instance, for some common types of decoherence processes (such as depolarizing and phase damping channels) 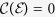 since they are unital quantum channels. However, for dissipative channels (e.g., amplitude damping channel) 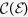 is exactly equal to the damping parameter *γ*, which can be thought of as the probability of losing a photon (here we adopt the notations in ref. 3). Intrudingly, the spontaneous emission process is one of the strongest cohering channels, due to the fact that it maps any state into a certain pure state (e.g., the ground state of a system). As a comparison, it’s worth noting that J. Wallman proposed a measure of coherence of quantum operations rather than the coherence of quantum states, where the role of purity is also highlighted^75^. Based on the commutator between quantum state and the elements of a specific symmetric group *G*, we formulate an asymmetry measure 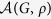 by integrating the Frobenius norm of the commutator over this group. Two distinct situations are considered: local *independent* and *collective* 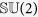 transformations. For *N*-qubit quantum states, 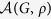 only relies on the purity and is actually equivalent to 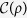 (or 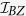) under local independent 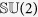 transformations. However, for local collective 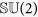 transformations, 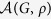 also depends on the quantum correlations between subsystems. One important open question is to find a closed expression of 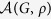 for arbitrary *N*-qubit states (especially *N* > 3) in this case.

## Methods

### Normalization of 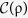 and 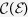


Here we only focus on 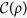, because 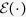 is a completely positive and trace preserving map and hence 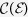 is also a valid density matrix. Trace-preserving property renders 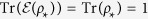, and we obtain


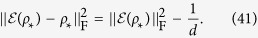


Note that for *p* ∈ [1, +∞) and an arbitrary *non-negative* vector 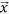, the following inequality holds for vector *p*-norms^76^


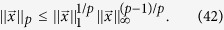


Since the Schatten-*p* norms of an operator *A* is equal to its vector *p*-norm of its singular values (e.g., 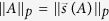), the above inequality also holds for Schatten-*p* norms. Therefore, we have


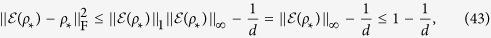


where we have used 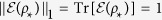 due to the positivity of 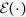 and the monotonicity 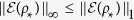. It is quite clear that the equality is satisfied only if the quantum channel 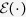 maps the maximally mixed state 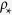 to a pure state, which only possesses intrinsic (e.g., basis-independent) coherence in our context.

### Necessity of unital channels

In ref. 70, it is proved that a dynamical semigroup is strictly purity-decreasing if and only if the Lindblad generator is unital. Here the quantum dynamic semigroup (in the Schrödinger picture) is a family of one-parameter linear trace-preserving maps 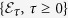 satisfying an additional property except for complete positivity, namely^77^


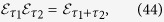


which is called semigroup condition or Markov property. However, we recall that a linear map 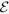 is completely positive *if and only if* it admits a Kraus representation. Thus in our context we are dealing with a more general class of dynamical maps. Note that Nielsen and Chuang stated in their book that a quantum process described in terms of an operator-sum representation cannot necessarily be written down as a master equation, especially for non-Markovian dynamics^3^. In particular, it is worth pointing out that the technique used in ref. 70 does not work for our purpose. Therefore, the necessity of unitality of operator-sum representation for strictly-decreasing purity is still an important unsolved problem.

Moreover, though we present an elegant proof of the sufficient part, we can reach this conclusion from a different perspective. For *α* ∈ (0, 1)∪(1, +∞), the quantum *α*-divergence^28^


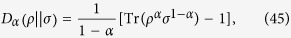


where we assume that supp(*ρ*)⊂supp(*σ*). The monotonicity of 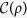 under unital channels follows from the facts that 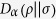 is monotone under completely positive and trace preserving maps in the range *α* ∈ (0, 2]^28^ and 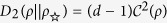.

### Integrals over independent and collective local unitary transformations

For independent local unitary transformations, the key task is to calculate the integral 
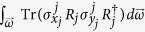
. For simplicity, we define 
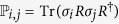
. A straightforward observation shows that the evaluations of 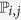 can be divided into four categories


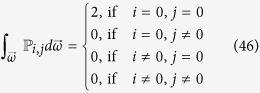


The first three situations are evident but the last one is not immediately obvious. In order to calculate 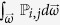, the key point is that for any *σ*_*j*_ (*j* = 1, 2, 3), one can always find an operator 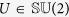 such that *Uσ*_*j*_*U*^†^ = *σ*_3_. Moreover, this operator can be absorbed into the average over 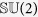. Therefore, we only need to consider 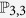. Finally, we can obtain


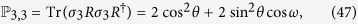


where we have used the identity


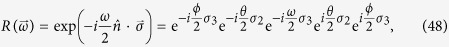


and the commutation relations


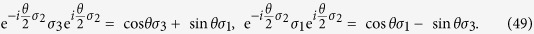


Finally, for any *i*, *j* = 1, 2, 3 we have


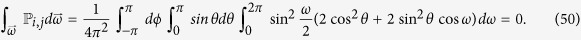


However, the situation is much more complex when confronted with the collective local unitary transformations. Take two-qubit state for example, and in this case we should deal with the integral 
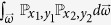
. Similar analysis leads to the fact


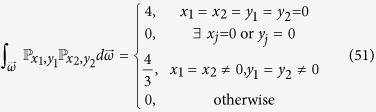


Special attention should be paid to the case *x*_1_ = *x*_2_ ≠ 0, *y*_1_ = *y*_2_ ≠ 0, that is


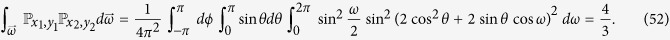


We have also considered the three-qubit state and only present the result here


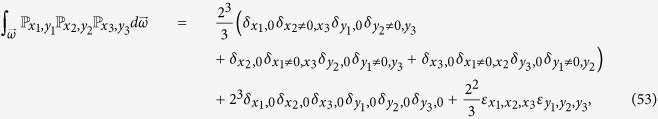


where *δ*_*i*,*j*_ and *ε*_*i*,*j*,*k*_ denote the Kronecker delta and Levi-Civita symbol, respectively. However, the generalization to arbitrary *N*-qubit states is still missing, which is left as an open question for further study.

## Additional Information

**How to cite this article**: Yao, Y. *et al*. Frobenius-norm-based measures of quantum coherence and asymmetry. *Sci. Rep.* **6**, 32010; doi: 10.1038/srep32010 (2016).
